# Next-generation sequencing using a targeted gene panel in advanced solid tumors: five years of experience from an Italian referral institution

**DOI:** 10.1007/s12094-026-04374-w

**Published:** 2026-06-03

**Authors:** Pasquale Pisapia, Antonino Iaccarino, Caterina De Luca, Francesco Pepe, Gianluca Russo, Mariantonia Nacchio, Domenica Di Giovanni, Domenico Cozzolino, Floriana Conticelli, Lucia Palumbo, Claudia Scimone, Gianluca Gragnano, Maria Russo, Maria Salatiello, Elena Vigliar, Claudio Bellevicine, Giancarlo Troncone, Umberto Malapelle

**Affiliations:** 1 Department of Public Health, University of Naples Federico II, via Sergio Pansini, 80131 Naples, Italy; 2 Department of Neurosciences, Reproductive and Odontostomatological Sciences, University of Naples Federico II, via Sergio Pansini, 80131 Naples, Italy

**Keywords:** NGS, Liquid biopsy, Molecular pathology, Molecular oncology, Targeted therapy, Biomarkers

## Abstract

**Aims:**

Next generation sequencing (NGS) represents the “holy grail” for the diagnostic algorithm for advanced solid tumors. In our experience, we commonly use our customized DNA-based NGS panel (namely, SiRe®). The aim of this study was to assess the efficiency of our customized DNA-based NGS panel in detecting molecular alterations in both tissue and liquid biopsy samples.

**Methods:**

We retrospectively retrieved molecular data from our electronic archives of advanced stage solid tumor cases tested by our DNA-based NGS approach from January 2018 to December 2022. Almost all samples (2045/2173, 94.1%), including liquid biopsies, were analyzed with our DNA-based NGS approach. We also retrieved all relevant molecular data on other tested biomarkers.

**Results:**

A total of n = 2173 advanced stage solid tumor patients were tested. Overall, at least one DNA-based alteration was detected in 52.7%, 66.6%, 87.6%, 68.8%, 46.7% of NSCLC ADC, CRC, GIST, melanoma, and breast cancer cases.

**Conclusions:**

The present study has provided a real-world practice experience on the efficiency of applying DNA-based NGS analysis to both tissue and liquid biopsy samples in detecting actionable mutations in advanced stage solid tumor patients.

**Supplementary Information:**

The online version contains supplementary material available at https://doi.org/10.1007/s12094-026-04374-w.

## Introduction

Cancer is the second leading cause of death worldwide, particularly among individuals younger than 85 years [1]. For most of the 20th century, chemo- and/or radiotherapy regimens were the only treatment options available for patients with unresectable solid tumors. However, with the advent of the 21st century, the concept of individualized treatment began to take hold as a way to address the limitations of traditional “one-size-fits-all” cancer therapies. Over the past two decades, personalized medicine has radically transformed the management and clinical outcomes of metastatic solid tumors, allowing clinicians to implement targeted therapies and immune checkpoint inhibitors (ICIs) [2–4].

In this rapidly evolving landscape, a multitude of clinically approved biomarkers—as well as many still under investigation—are being tested in clinical trials involving patients with advanced or metastatic solid tumors [5]. Consequently, molecular profiling has become essential for determining patient eligibility for individualized treatments.

Currently, histological and cytological tissue specimens remain the “gold standard” materials for obtaining tumor-derived nucleic acids for molecular testing [6]. However, in patients with advanced-stage disease, molecular testing is often hindered by the limited availability—or complete absence—of neoplastic tissue [7]. In this context, liquid biopsy has emerged as a major breakthrough in diagnostic oncology, enabling an increasing number of patients with advanced-stage disease to benefit from targeted therapies and immunotherapy [8]. Despite its high sensitivity as an alternative molecular testing tool, liquid biopsy still presents challenges for molecular pathologists, who must analyze an ever-growing number of emerging biomarkers across multiple solid tumor types.

In this scenario, the application of next-generation sequencing (NGS) to both tissue and liquid biopsy samples represents the “holy grail” of diagnostic algorithms for advanced solid tumors [9]. In our institution, we routinely employ a customized DNA-based NGS panel (SiRe®), which has proven to be highly efficient in detecting actionable biomarkers in both tissue and liquid biopsy specimens from patients with advanced-stage solid tumors [10].

In the present study, to further validate our in-house NGS panel, we retrospectively analyzed data from our archives of patients with advanced-stage solid tumors who were referred to our laboratory for molecular testing between January 2018 and December 2022. Almost all samples, including liquid biopsies, were analyzed using our DNA-based NGS approach.

## Materials and methods

### Study design

**Fig. 1:**
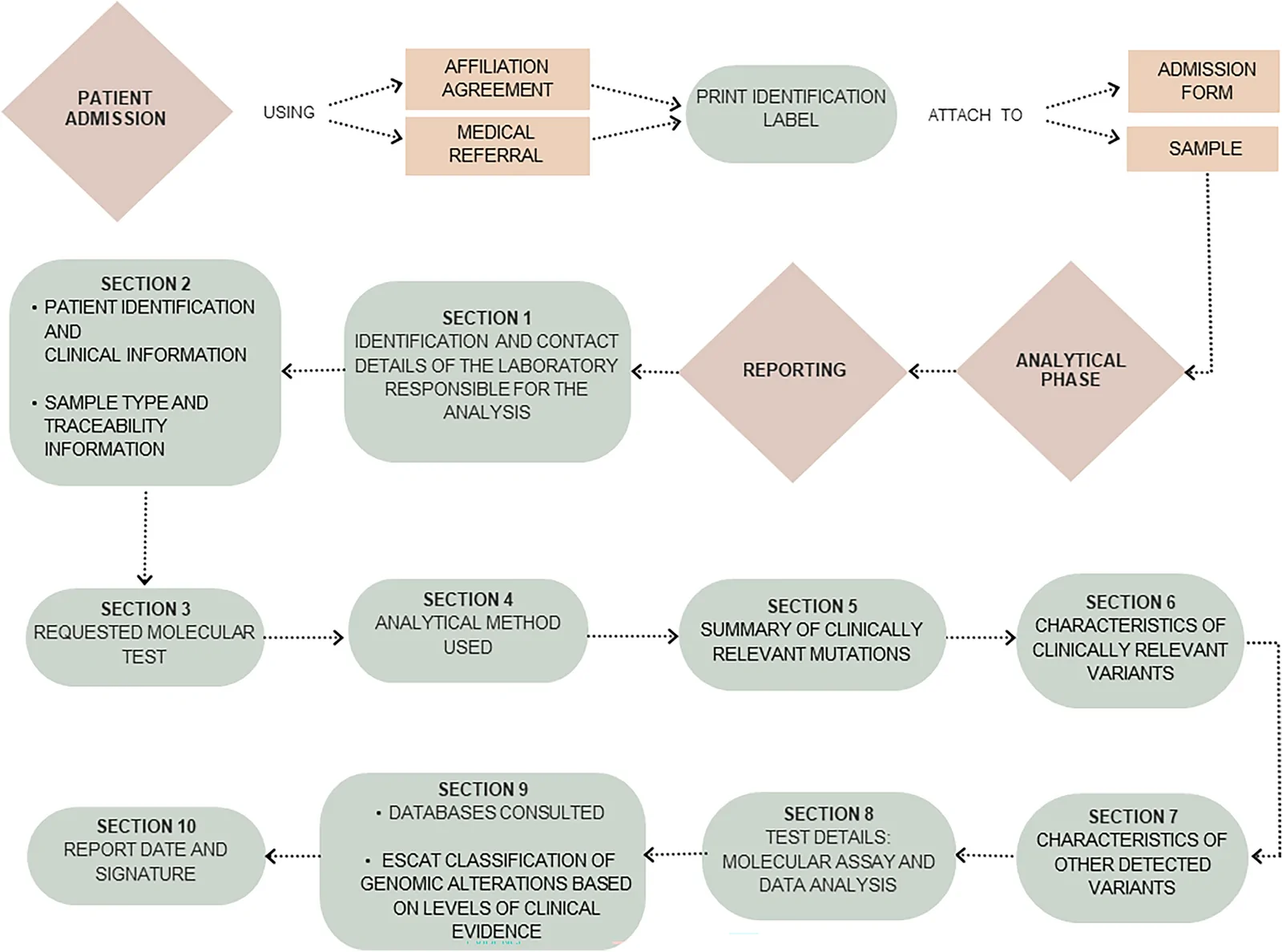
Laboratory workflow

**Fig. 2:**
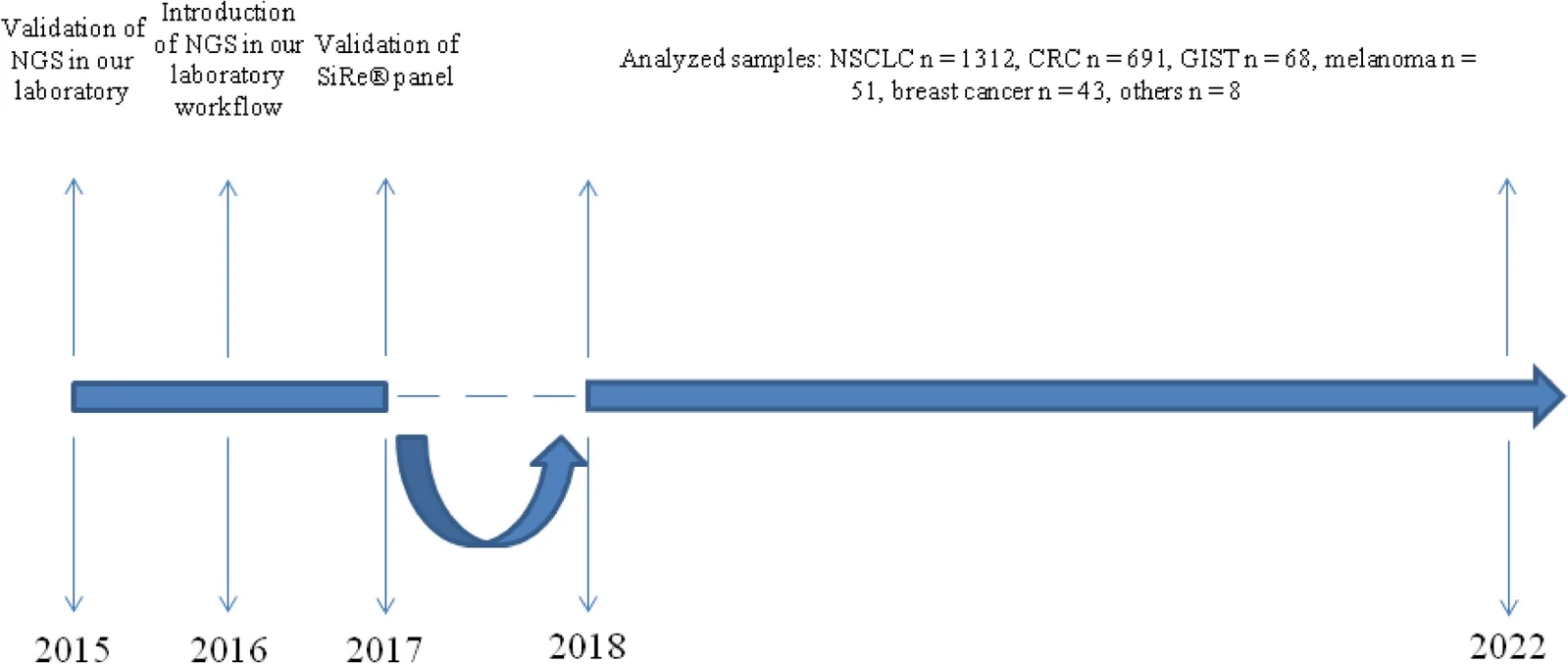
Roadmap of the adoption of NGS in our laboratory: from the validation against Sanger sequencing in 2015, the introduction in the routine diagnostic workflow, the validation of SiRe® panel, to the routine adoption of this latter panel from January 2018 to December 2022

We retrospectively retrieved molecular data from our electronic archives of advanced stage solid tumor cases tested by our DNA-based NGS approach from January 2018 to December 2022. We also retrieved all relevant molecular data on other tested biomarkers. Liquid biopsy and tissue analyses were performed on independent patient cohorts, with no matched samples. Moreover, patients analyzed at baseline were different from those analyzed at resistance. Figures 1 and 2 illustrate the laboratory workflow.

Institutional Review Board approval was waived since this study was retrospective and, thus, required no deviation from the standard of care. Written informed consent was obtained from all the patients in accordance with the general authorization for processing personal data for scientific research purposes from “The Italian Data Protection Authority” (http://www.garanteprivacy.it/web/guest/home/docweb/-/docwebdisplay/export/2485392). All information regarding human material was managed using anonymous numerical codes, and all samples were handled in compliance with the Declaration of Helsinki (https://www.wma.net/policies-post/wma-declaration-of-helsinki-ethical-principles-for-medical-research-involving-human-subjects/).

### SiRe® molecular analysis and other molecular assays

DNA-based NGS (SiRe®) analysis was carried out as previously described. Briefly, this panel, which was designed, developed, and validated in the Molecular Predictive Pathology Laboratory of the Department of Public Health at the University of Naples Federico II, is able to cover mutations within seven genes (Epidermal Growth Factor Receptor [*EGFR*], Kirsten Rat Sarcoma Viral Oncogene Homolog [*KRAS*], V-Raf Murine Sarcoma Viral Oncogene Homolog B [*BRAF*], Neuroblastoma RAS Viral Oncogene Homolog [*NRAS*], KIT Proto-Oncogene, Receptor Tyrosine Kinase [*KIT*], Platelet Derived Growth Factor Receptor Alpha [*PDGFRα*], and Phosphatidylinositol-4,5-Bisphosphate 3-Kinase Catalytic Subunit Alpha [*PIK3CA*]) [10, 11]. In particular, only variants with ≥ 1% (tissue samples) and 0.02% (liquid biopsy samples) allelic frequency and a quality score ≥ 20, within an amplicon that covered at least 1000X alleles, were called (Supplementary Tables 1 and 2).

Regarding tissue samples, only specimens with at least 5% of tumor cell content were tested.

RNA-based NGS analysis was carried out with the SiRe fusion panel as previously described. Briefly, this panel, which, like the previous one, was designed, developed, and validated in the Molecular Predictive Pathology Laboratory of the Department of Public Health at the University of Naples Federico II, was able to cover gene fusions within Anaplastic Lymphoma Receptor Tyrosine Kinase (*ALK*), ROS Proto-Oncogene 1, Receptor Tyrosine Kinase (*ROS1*), Rearranged During Transfection (*RET*), Neurotrophic Receptor Tyrosine Kinase (*NTRK*), as well as MET Proto-Oncogene, Receptor Tyrosine Kinase (*MET*) exon 14 skipping [12].

Real**-**time polymerase chain reaction (RT-qPCR) for *ALK*, *ROS1*, *RET*, *NTRK* gene rearrangements, and *MET* exon 14 skipping was performed with the Easy PGX platform (Diatech Pharmacogenetics, Jesi, Italy), as previously reported [12].

Immunohistochemistry/immunocytochemistry (IHC/ICC) analysis for ALK, ROS1, and Programmed Death-Ligand 1 (PD-L1) expression was performed as previously described. More in detail, the Ventana D5F3 companion diagnostic (CDx) assay (Ventana Medical Systems, Oro Valley, AZ, USA) and D4D6 clones (Cell Signaling Technology, Inc., Danvers, MA, USA) were employed for ALK and ROS1 evaluation, respectively. PD-L1 expression was evaluated with a validated laboratory developed test (LDT); this test uses either the 22C3 anti-PD-L1 primary antibody concentrate (Dako) on the Ventana BenchMark XT platform, or the SP263 companion diagnostic kit (Ventana Medical Systems) [13].

Microsatellite instability (MSI) was detected with the Bethesda panel of 5 biomarkers (BAT25, BAT26, D2, D5 and D17), as previously described [14].

## Results

**Fig. 3:**
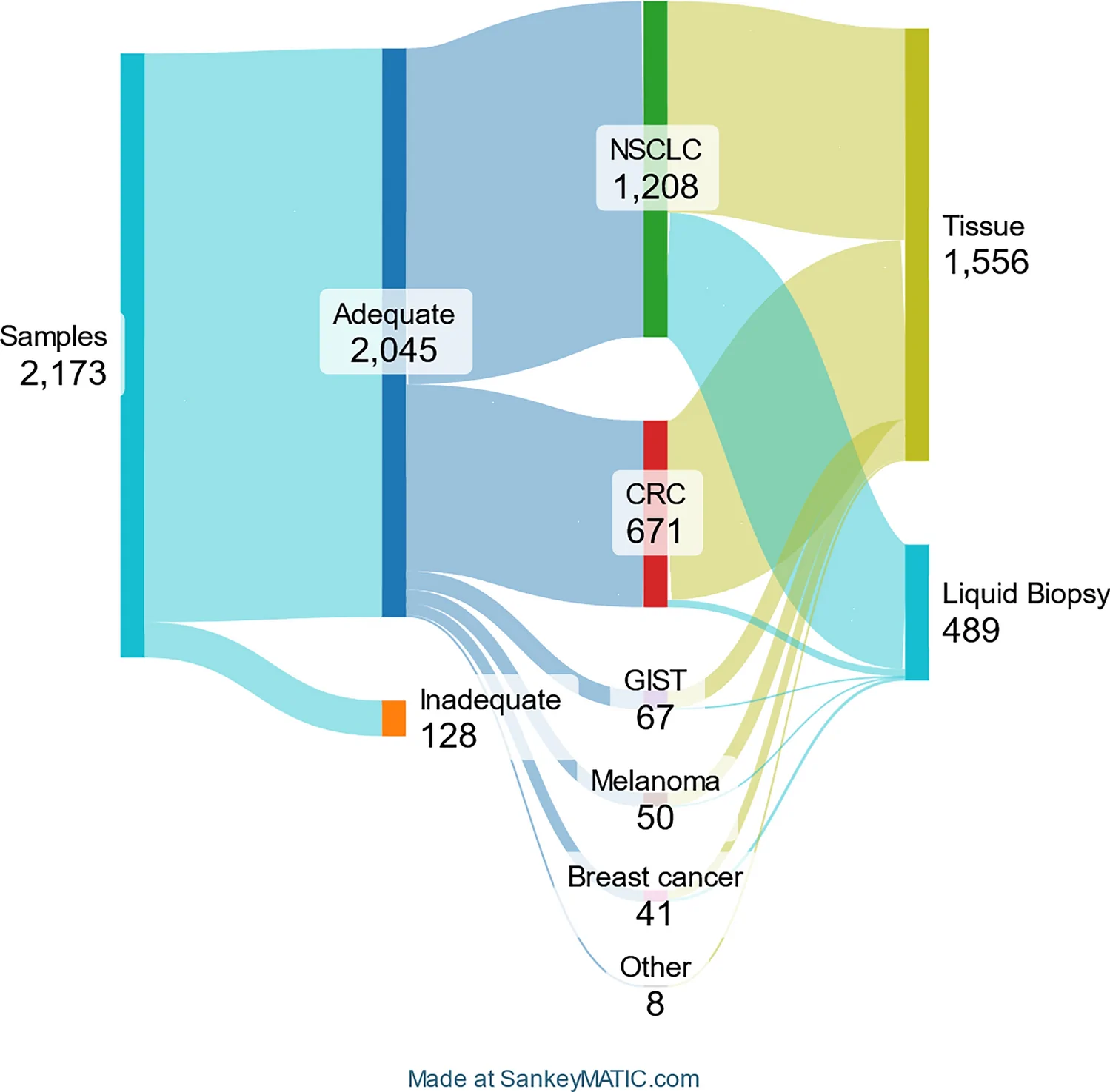
Sankey diagram summarizing results of SiRe® panel

**Fig. 4:**
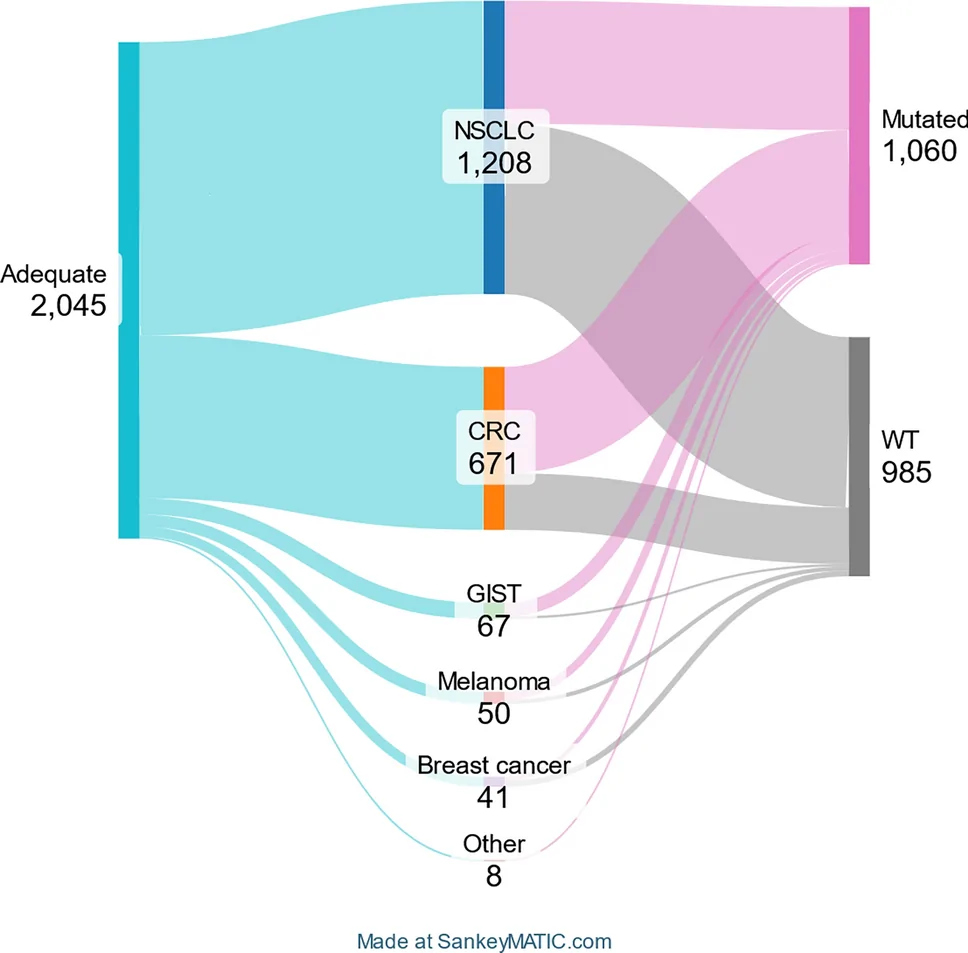
Sankey diagram summarizing more in details results of SiRe® panel

A total of n = 2173 advanced stage solid tumor patients were tested by our DNA-based NGS SiRe® panel from January 1th, 2018 to December 31th, 2022. Remarkably, our overall failure rate due to rejected samples was as low as 5.9% (128/2173). In all instances, this was due to low tumor cell content or insufficient nucleic acid quality or quantity. As expected, the vast majority of the analyzed samples belonged to patients with non-small cell lung cancer (NSCLC) (60.4%, 1312/2137), followed by those with colo-rectal cancer (CRC) (31.8%, 691/2173), gastrointestinal stromal tumor (GIST) (3.1%, 68/2173), melanoma (2.3%, 51/2173), breast cancer (2.0, 43/2173), and others (0.4%, 8/2173) (Figures 3 and 4 and Supplementary Table 3).

### NSCLC

Overall, 60.4% (1312/2137) of tested samples were from advanced-stage NSCLC patients, and our DNA-based SiRe® NGS panel successfully analyzed 92.1% (1208/1312) of these samples. Most specimens were tissue samples (63.0%, 761/1208), while 37.0% (447/1208) were liquid biopsies. Among tissue samples, histological specimens predominated (59.5%, 453/761), mainly biopsies (74.8%, 339/453), whereas surgical resections accounted for 25.2% (114/453); cytological samples represented 40.5% (308/761), with cell blocks being the most common preparation (73.4%, 226/308).

Nearly all liquid biopsy samples were plasma (99.4%, 444/447), with pleural effusions (0.4%, 2/447) and cerebrospinal fluid (0.2%, 1/447) being rare. Most liquid biopsies were collected at baseline (61.3%, 274/447), and the remainder after acquired resistance to EGFR TKI therapy (38.7%, 173/447). Testing for additional biomarkers varied: *ALK* (52.7%), *ROS1* (50.8%), PD-L1 (50.7%), *RET*/*MET* exon 14 skipping/*NTRK* (15.6%), and MSI (0.1%).

Regarding morphological diagnosis, NSCLC adenocarcinoma/favor adenocarcinoma was most frequent (87.5%, 1056/1208), followed by NSCLC not otherwise specified (NOS) (5.7%, 69/1208), NSCLC squamous/favor squamous (5.5%, 67/1208), NSCLC adenosquamous (1.1%, 13/1208), and NSCLC large cell neuroendocrine/favor LCNEC (0.2%, 3/1208).

### NSCLC ADC/favor ADC

The NGS SiRe® panel successfully analyzed 87.5% (1,056/1,208) of advanced-stage NSCLC ADC/favor ADC samples. Among adequate specimens, 58.1% (614/1,056) were tissue and 41.9% (442/1,056) liquid biopsies. Most liquid biopsies were collected at baseline (60.9%, 269/442), with the remainder obtained after acquired resistance to EGFR-TKI therapy (39.1%, 173/442).

In tissue samples, at least one DNA-based alteration was detected in 52.7% (323/614) of cases. Single mutations included *KRAS* (32.5%), *EGFR* (12.1%), *BRAF* (4.2%), *PIK3CA* (1.0%), and *NRAS* (0.7%). Co-occurring mutations were rare (< 1% overall).

Among other biomarkers, 79.9% (554/693), 77.2% (535/693), 76.0% (527/693), and 23.9% (166/693) of cases were referred for *ALK*, *ROS1*, PD-L1, and *RET*/*MET* exon 14/*NTRK* testing, respectively, with adequacy rates above 94% for all assays. ALK and ROS1 were mainly tested by IHC/ICC (≈68%), identifying 3.6% and 0.8% positive cases, respectively. *RET*/*NTRK*/*MET* were primarily analyzed by NGS (97.6%), revealing 8 *RET*-rearranged cases (4.8%), including one with concurrent *PIK3CA* p.E545K (Table 1).

PD-L1 expression (≥1%) was detected in 46.8% (232/496) of tested samples—22.6% with low (1–49%) and 24.2% with high (≥50%) expression. *KRAS* mutations were the most frequent co-alterations in both PD-L1 categories.

In baseline liquid biopsies (n = 269), 32.3% harbored at least one alteration, most commonly *KRAS* (11.5%) and *EGFR* (7.9%). No rearrangements or *MET* exon 14 skipping were detected (Table 2).

In liquid biopsies from patients with acquired TKI resistance (n = 173), 46.9% carried at least one alteration—predominantly *EGFR* (41.6%)—while *PIK3CA* and *KRAS* mutations occurred infrequently. Co-occurring mutations were rare, and no fusions or *MET* exon 14 alterations were observed (Table 2).

### NSCLC NOS

The SiRe® panel successfully analyzed 5.7% (69/1,208) of advanced-stage NSCLC samples classified as NOS. Almost all specimens were tissue samples (98.6%, 68/69); only one liquid biopsy (1.4%), collected at baseline, was analyzed and showed no detectable alterations. Among the 68 adequate tissue samples, at least one DNA-based alteration was identified in 42.6% (29/68) of cases. The most frequent mutations involved *KRAS* (25.0%), followed by *EGFR* (8.8%), *BRAF* (5.9%), and *NRAS* (2.9%) (Table 1).

**Table 1:** SiRe® results on NSCLC tissue samples

Diagnosis (total, %)	NSCLC ADC/favor ADC (1056, 87.5)	n, %	NSCLC NOS (69, 5.7)	n, %	NSCLC SqCC/favor SqCC (67, 5.5)	n, %	NSCLC adenosquamous (13, 1.1)	n, %	NSCLC LCNEC/favor LCNEC (3, 0.2)	n, %
SiRe® adequately tested tissue samples	SiRe® adequately tested tissue samples	614, 58.1	SiRe® adequately tested tissue samples	68, 98.6	SiRe® adequately tested tissue samples	64, 95.5	SiRe® adequately tested tissue samples	13, 100.0	SiRe® adequately tested tissue samples	2, 66.7
*KRAS*	Total mutations	206, 33.5	Total mutations	17, 25.0	Total mutations	2, 3.1	Total mutations	0, 0.0	Total mutations	0, 0.0
	Alone	200, 32.5	Alone	17, 25.0	Alone	2, 3.1				
	Co-occurring mutations with other tested genes	6, 1.0								
*KRAS* mutations	Exon 2 non p.G12C	100, 48.5	Exon 2 non p.G12C	7, 41.2	Exon 2 non p.G12C	1, 50.0	–	–	–	–
	Exon 2 p.G12C	90, 43.7	Exon 2 p.G12C	8, 47.1	Exon 2 p.G12C	1, 50.0				
	Exon 3	13, 6.3	Exon 3	2, 11.7						
	Exon 4	2, 1.0								
	Co-mutations	1, 0.5								
*EGFR*	Total mutations	81, 13.2	Total mutations	6, 8.8	Total mutations	4, 6.3	Total mutations	3, 23.1	Total mutations	0, 0.0
	Alone	74, 12.1	Alone	6, 8.8	Alone	4, 6.3	Alone	3, 23.1		
	Co-occurring mutations with other tested genes	7, 1.1								
*EGFR* mutations	Exon 18	3, 3.7	Exon 18	1, 16.7	Exon 18	1, 25.0	Exon 19 deletions	2, 66.7	–	–
	Exon 19 deletions	29, 35.8	Exon 19 deletions	4, 66.6	Exon 19 deletions	2, 50.0	Exon 21	1, 33.3		
	Exon 20 point mutations	3, 3.7	Exon 21	1, 16.7	Exon 21	1, 25.0				
	Exon 20 insertions	11, 13.6								
	Exon 21	28, 34.6								
	Compound	7, 8.6								
*BRAF*	Total mutations	31, 5.0	Total mutations	4, 5.9	Total mutations	1, 1.6	Total mutations	0, 0.0	Total mutations	0, 0.0
	Alone	26, 4.2	Alone	4, 5.9	Alone	1, 1.6				
	Co-occurring mutations with other tested genes	5, 0.8								
*BRAF* mutations	Exon 11	12, 38.7	Exon 11	2, 50.0	Exon 11	1, 100.0	–	–	–	–
	Exon 15 non p.V600E	7, 22.6	Exon 15 non p.V600E	2, 50.0						
	Exon 15 p.V600E	12, 38.7								
*PIK3CA*	Total mutations	13, 1.8	Total mutations	0, 0.0	Total mutations	2, 3.1	Total mutations	0, 0.0	Total mutations	0, 0.0
	Alone	6, 0.7			Alone	2, 3.1				
	Co-occurring mutations with other tested genes	7, 1.1								
*PIK3CA* mutations	Exon 9	10, 76.9	–	–	Exon 9	2, 100.0	–	–	–	–
	Exon 20	3, 23.1								
*NRAS*	Total mutations	5, 0.9	Total mutations	2, 2.9	Total mutations	1, 1.6	Total mutations	1, 7.7	Total mutations	0, 0.0
	Alone	4, 0.7	Alone	2, 2.9	Alone (1, 1.6)	1, 1.6	Alone	1, 7.7		
	Co-occurring mutations with other tested genes	1, 0.2								
*NRAS* mutations	Exon 2	3, 60.0	Exon 2	1, 50.0	Exon 3	1, 100.0	Exon 3	1, 100.0	–	–
	Exon 3	2, 40.0	Exon 3 (1, 50.0)	1, 50.0						
*KIT*	Total mutations	0, 0.0	Total mutations	0, 0.0	Total mutations	0, 0.0	Total mutations	0, 0.0	Total mutations	0, 0.0
*KIT* mutations	–	–	–	–	–	–	–	–	–	–
*PDGFRα*	Total mutations	0, 0.0	Total mutations	0, 0.0	Total mutations	0, 0.0	Total mutations	0, 0.0	Total mutations	0, 0.0
*PDGFRα* mutations	–	–	–	–	–	–	–	–	–	–

ADC Adenocarcinoma, *BRAF* V-Raf murine sarcoma viral oncogene homolog B, *EGFR* Epidermal growth factor receptor, *KIT* KIT proto-oncogene, receptor tyrosine kinase, *KRAS* Kirsten rat sarcoma viral oncogene homolog, LCNEC Large cell neuroendocrine carcinoma, *n* Number, *NOS* Not otherwise specified, *NRAS* Neuroblastoma RAS viral oncogene homolog, NSCLC Non-small cell lung cancer, *PDGFRα* Platelet derived growth factor receptor alpha, *PIK3CA* Phosphatidylinositol-4,5-bisphosphate 3-kinase catalytic subunit alpha, *SqCC* Squamous cell carcinoma

Regarding other biomarkers, 82.1%, 79.5%, 79.5%, and 19.2% of patients were referred for *ALK*, *ROS1*, PD-L1, and *RET*/*MET* exon 14 skipping/*NTRK* testing, respectively. Adequate analysis rates were high: 90.6%, 90.3%, 83.9%, and 100.0%. ALK and ROS1 were mainly assessed by IHC/ICC (≈73%), with complementary testing by the SiRe® fusion NGS panel. No *ALK* rearrangements were found, whereas one *ROS1*-positive case (1.8%) also harbored a *KRAS* p.G12V mutation. Among *RET*/*NTRK*/*MET* analyses, one *MET* exon 14 skipping alteration (6.7%) was identified.

PD-L1 expression (≥ 1%) was observed in 48.1% (25/52) of evaluable samples, including 17.3% with low (1–49%) and 30.8% with high (≥ 50%) expression. Low PD-L1 positivity was mainly associated with *KRAS*, *EGFR*, and *NRAS* mutations, while high expression correlated more frequently with *KRAS*, and occasionally with *EGFR*, *MET* exon 14 skipping, and *KRAS* + *ROS1* co-alterations.

### NSCLC SqCC/favor SqCC

The SiRe® panel successfully analyzed 5.5% (67/1,208) of advanced-stage NSCLC samples classified as SqCC/favor SqCC. Most specimens were tissue samples (95.5%, 64/67), while three liquid biopsies (4.5%) collected at baseline showed no detectable alterations. Among the 64 adequate tissue samples, 15.7% (10/64) harbored at least one DNA-based alteration. Mutations involved *EGFR* (6.3%), *KRAS* (3.1%), *PIK3CA* (3.1%), *BRAF* (1.6%), and *NRAS* (1.6%) (Table 1).

Regarding other biomarkers, 65.8%, 60.5%, 80.3%, and 17.1% of patients were referred for *ALK*, *ROS1*, PD-L1, and *RET*/*MET* exon 14 skipping/*NTRK* testing, respectively. Detection success rates were high—96.0%, 95.7%, 95.1%, and 100.0%. *ALK* and *ROS1* were mainly tested by IHC/ICC (~71%), followed by NGS, and no rearrangements were detected. *RET*/*NTRK*/*MET* were analyzed exclusively by NGS, with no alterations identified.

PD-L1 expression (≥ 1%) was observed in 58.6% (34/58) of evaluable samples, including 36.2% with low (1–49%) and 22.4% with high (≥ 50%) expression. Low PD-L1 positivity was associated with *KRAS* and *PIK3CA* mutations (each 4.8%), whereas high expression was linked to *EGFR* in one case (7.7%).

### NSCLC adenosquamous

The SiRe® panel successfully analyzed 1.1% (13/1,208) of advanced-stage NSCLC samples classified as adenosquamous carcinoma. All analyzed specimens were tissue samples. Among these, 30.8% (4/13) harbored at least one DNA-based alteration, involving *EGFR* (23.1%) and *NRAS* (7.7%) (Table 1).

Regarding other biomarkers, 87.5%, 87.5%, 81.3%, and 18.8% of patients were referred for *ALK*, *ROS1*, PD-L1, and *RET*/*MET* exon 14 skipping/*NTRK* testing, respectively, with high detection success rates (92.9%, 92.9%, 100.0%, and 100.0%). ALK and ROS1 were primarily analyzed by IHC/ICC (~77%) and secondarily by NGS, with no rearrangements detected. *RET*/*NTRK*/*MET* were exclusively analyzed by NGS, identifying one RET rearranged case.

PD-L1 expression (≥ 1%) was observed in 69.2% (9/13) of samples: 23.1% showed low (1–49%) and 46.1% high (≥ 50%) expression. Low PD-L1 positivity was associated with *EGFR* mutation (33.3%), whereas high expression was linked to *NRAS* (25%).

### NSCLC LCNEC/favor LCNEC

Overall, the SiRe® panel correctly analyzed 0.2% (3/1208) of NSCLC samples from advanced stage NSCLC LCNEC/favor LCNEC patients. Most specimens were tissue samples (2/3, 66.7%); in all instances, no DNA- or and RNA-based alterations, or PD-L1 expression levels were detected (Table 1). The remaining specimen was a liquid biopsy sample (1/3, 33.3%) featuring a *KRAS* exon 2 mutation.

### CRC

Using the SiRe® panel, a total of 691 metastatic colorectal cancer (mCRC) samples (31.8%) were analyzed, achieving a 97.1% success rate (671/691). Most analyzed specimens were tissue samples (96.1%, 645/671), while plasma samples accounted for 3.9% (26/671). Among tissue samples, nearly all were histological (99.1%, 639/645), mainly from surgical resections (72.3%) rather than biopsies (27.7%). Cytological specimens represented 0.9% (6/645), predominantly cell blocks (83.3%).

The Bethesda panel was additionally used in 366/645 cases (56.7%) for MSI testing, with a 98.9% success rate (362/366). Only a few cases (0.5%, 3/645) were referred for *ALK*, *ROS1*, and *RET*/*MET* exon 14 skipping/*NTRK* analysis (Table 3).

**Table 2:** SiRe® results on NSCLC ADC/favor ADC liquid biopsy samples

Basal setting: total (%)	269 (25.5)	n, %	Resistance setting: total (%)	173 (16.4)	n, %
*KRAS*	Total mutations	31, 11.5	*KRAS*	Total mutations	3, 1.7
	Alone	31, 11.5		Alone	2, 1.2
				Co-occurring mutations with other tested genes	1, 0.5
*KRAS* mutations	Exon 2 non p.G12C	16, 51.6	*KRAS* mutations	Exon 2 non p.G12C	3, 100.0
	Exon 2 p.G12C	13, 41.9			
	Exon 3	2, 6.5			
*EGFR*	Total mutations	23, 8.6	*EGFR*	Total mutations	75, 43.3
	Alone	21, 7.9		Alone	72, 41.6
	Co-occurring mutations with other tested genes	1, 0.7		Co-occurring mutations with other tested genes	3, 1.7
*EGFR* mutations	Exon 18	2, 8.7	*EGFR* mutations	Exon 18	1, 1.3
	Exon 19 deletions	16, 69.6		Exon 19 deletions	25, 33.3
	Exon 20 insertions	2, 8.7		Exon 20 point mutations	3, 4.0
	Exon 21	2, 8.7		Exon 21	12, 16.0
	Compound	1, 4.3		Compound	34, 45.4
*BRAF*	Total mutations	2, 0.7	*BRAF*	Total mutations	0, 0.0
	Alone	2, 0.7			
*BRAF* mutations	Exon 15 non p.V600E	1, 50.0	*BRAF* mutations	–	–
	Exon 15 p.V600E	1, 50.0			
*PIK3CA*	Total mutations	3, 1.1	*PIK3CA*	Total mutations	6, 3.5
	Alone	1, 0.4		Alone	4, 2.3
	Co-occurring mutations with other tested genes	2, 0.7		Co-occurring mutations with other tested genes	2, 1.2
*PIK3CA* mutations	Exon 9	2, 66.7	*PIK3CA* mutations	Exon 9	3, 50.0
	Exon 20	1, 33.3		Exon 20	3, 50.0
*NRAS*	Total mutations	2, 0.7	*NRAS*	Total mutations	0, 0.0
	Alone	2, 0.7			
*NRAS* mutations	Exon 2	1, 50.0	*NRAS* mutations	–	–
	Exon 3	1, 50.0			
*KIT*	Total mutations	0, 0.0	*KIT*	Total mutations	0, 0.0
*KIT* mutations	–	–	*KIT* mutations	–	–
*PDGFRα*	Total mutations	1, 0.4	*PDGFRα*	Total mutations	0, 0.0
	Alone	1, 0.4			
*PDGFRα* mutations	Exon 18	1, 100.0	*PDGFRα* mutations	–	–

*ADC* Adenocarcinoma, *BRAF* V-Raf murine sarcoma viral oncogene homolog B, *EGFR* Epidermal growth factor receptor, *KIT* KIT proto-oncogene, receptor tyrosine kinase, *KRAS* Kirsten rat sarcoma viral oncogene homolog, *n* Number, *NRAS* Neuroblastoma RAS viral oncogene homolog, NSCLC Non-small cell lung cancer, *PDGFRα* Platelet derived growth factor receptor alpha, *PIK3CA* Phosphatidylinositol-4,5-bisphosphate 3-kinase catalytic subunit alpha

Among all adequately tested tissue samples (n = 645), 66.6% (429/645) harbored at least one DNA-based alteration. Single mutations included *KRAS* (37.7%), *PIK3CA* (5.6%), *BRAF* (5.6%), *NRAS* (5.1%), and *KIT* (0.2%). *PIK3CA* frequently co-occurred with other alterations, most commonly *KRAS* (9.3%), *BRAF* (1.4%), and less frequently *NRAS*, *EGFR*, and combined profiles such as *EGFR* + *KRAS* or *KRAS* + *NRAS* (each <0.3%). Additional rare combinations included *KRAS* + *BRAF* (0.3%) and *BRAF* + *NRAS* (0.2%) (Table 3).

**Table 3:** SiRe® results on CRC tissue samples

N (%)	671 (100.0)	n, %
SiRe® adequately tested tissue samples	SiRe® adequately tested tissue samples	645, 97.1
*KRAS*	Total mutations	309, 47.9
	Alone	243, 37.7
	Co-occurring mutations with other tested genes	66, 10.2
*KRAS* mutations	Exon 2 non p.G12C	253, 81.9
	Exon 2 p.G12C	22, 7.1
	Exon 3	13, 4.2
	Exon 4	14, 4.5
	Co-mutations	7, 2.3
*EGFR*	Total mutations	4, 0.6
	Alone	0, 0.0
	Co-occurring mutations with other tested genes	4, 0.6
*EGFR* mutations	Exon 18	2, 50.0
	Exon 19 deletions	1, 25.0
	Compound	1, 25.0
*BRAF*	Total mutations	50, 7.7
	Alone	37, 5.7
	Co-occurring mutations with other tested genes	13, 2.0
*BRAF* mutations	Exon 11	2, 4.0
	Exon 15 non p.V600E	5, 10.0
	Exon 15 p.V600E	43, 86.0
*PIK3CA*	Total mutations	110, 17.0
	Alone	37, 5.7
	Co-occurring mutations with other tested genes	73, 11.3
*PIK3CA* mutations	Exon 2	5, 4.5
	Exon 7	9, 8.2
	Exon 9	63, 57.3
	Exon 20	30, 27.3
	Co-mutations	3, 2.7
*NRAS*	Total mutations	37, 5.7
	Alone	33, 5.1
	Co-occurring mutations with other tested genes	4, 0.6
*NRAS* mutations	Exon 2	13, 35.1
	Exon 3	24, 64.9
*KIT*	Total mutations	1, 0.2
	Alone	1, 0.2
*KIT* mutations	Exon 13	1, 100.0
*PDGFRα*	Total mutations	0, 0.0
*PDGFRα* mutations	–	–

*BRAF* V-Raf murine sarcoma viral oncogene homolog B, CRC Colo-rectal cancer, *EGFR* Epidermal growth factor receptor, *KIT* KIT proto-oncogene, receptor tyrosine kinase, *KRAS* Kirsten rat sarcoma viral oncogene homolog, *n* Number, *NRAS* Neuroblastoma RAS viral oncogene homolog, *PDGFRα* Platelet derived growth factor receptor alpha, *PIK3CA* Phosphatidylinositol-4,5-bisphosphate 3-kinase catalytic subunit alpha

Finally, one of the three cases tested with the SiRe® fusion panel showed a *KIF5B*(16)::*RET*(12) rearrangement.

Among the 347 patients tested for MSI, 9.2% (32/347) were MSI-high (MSI-H). MSI-H occurred alone in 28.2% (9/32) of cases and co-occurred with *KRAS* (53.1%, 17/32), *PIK3CA* (9.4%, 3/32), *BRAF* (3.1%, 1/32), *KRAS* + *PIK3CA* (3.1%, 1/32), or *BRAF* + *PIK3CA* (3.1%, 1/32).

Among 26 liquid biopsy samples, 9 patients (34.5%) harbored mutations, including *KRAS* (15.4%, 4/26), *NRAS* (11.5%, 3/26), *PIK3CA* (3.8%, 1/26), and *KRAS* + *BRAF* (3.8%, 1/26).

### GIST

Using the SiRe® panel, 68 (3.1%) GIST samples were tested, with a success rate of 98.5% (67/68). Most specimens were tissue samples (95.5%, 64/67), primarily histological (98.4%, 63/64), including surgical resections (87.3%, 55/63) and biopsies (12.7%, 8/63); a small fraction were cytological cell blocks (1.6%, 1/64) or plasma samples (4.5%, 3/64).

Among the 64 adequate tissue samples, 87.6% (56/64) harbored at least one DNA-based alteration. KIT mutations were the most frequent (75.0%, 48/64), followed by *PDGFRα* (9.4%, 6/64) and *BRAF* (1.6%, 1/64). A single case (0.2%) showed a co-occurring *EGFR* + *KIT* mutation (Table 4).

Regarding the liquid biopsy samples (n = 3), only n = 1 (33.3%) exon 18 *PDGFRα* mutation was reported.

### Melanoma

Overall, the SiRe® panel analyzed 51 (2.3%) melanoma samples, achieving a 98.0% (50/51) success rate. Most specimens were tissue samples (96.0%, 48/50), primarily histological (91.7%, 44/48), with surgical resections (84.1%, 37/44) more frequent than biopsies (15.9%, 7/44). Cytological specimens accounted for 8.3% (4/48), evenly split between cell blocks and smears. Two samples (4.0%) were plasma-based liquid biopsies.

**Table 4:** SiRe® results on GIST tissue samples

N (%)	67 (100.0)	n, %
SiRe® tested tissue samples	SiRe® tested tissue samples	64, 95.5
*KRAS*	Total mutations	0, 0.0
*KRAS* mutations	–	–
*EGFR*	Total mutations	1, 1.6
	Alone	0, 0.0
	Co-occurring mutations with other tested genes	1, 1.6
*EGFR* mutations	Compound	1, 100.0
*BRAF*	Total mutations	1, 1.6
	Alone	1, 1.6
*BRAF* mutations	Exon 15 p.V600E	1, 100.0
*PIK3CA*	Total mutations	0, 0.0
*PIK3CA* mutations	–	–
*NRAS*	Total mutations	0, 0.0
*NRAS* mutations	–	–
*KIT*	Total mutations	49, 76.6
	Alone	48, 75.0
	Co-occurring mutations with other tested genes	1, 1.6
*KIT* mutations	Eexon 9	8, 16.3
	Exon 11	41, 83.7
*PDGFRα*	Total mutations	6, 9.4
	Alone	6, 9.4
*PDGFRα* mutations	Exon 18	6, 100.0

*BRAF* V-Raf murine sarcoma viral oncogene homolog B, *EGFR* Epidermal growth factor receptor, GIST Gastrointestinal stromal tumor, *KIT* KIT proto-oncogene, receptor tyrosine kinase, *KRAS* Kirsten rat sarcoma viral oncogene homolog, *n* Number, *NRAS* Neuroblastoma RAS viral oncogene homolog, *PDGFRα* platelet derived growth factor receptor alpha, *PIK3CA* phosphatidylinositol-4,5-bisphosphate 3-kinase catalytic subunit alpha

Among the 48 adequate tissue samples, 68.8% (33/48) harbored at least one DNA-based alteration. *BRAF* (29.2%, 14/48) and *NRAS* (25.0%, 12/48) were the most common, followed by *KIT* (4.2%, 2/48) and *KRAS* (2.0%, 1/48). Concomitant mutations were rare: *NRAS* + *BRAF* in 4.2% (2/48), *BRAF* + *KIT* in 2.1% (1/48), and *BRAF* + *PIK3CA* in 2.1% (1/48). Results are summarized in Table 5.

**Table 5:** SiRe® results on melanoma tissue samples

N (%)	50 (100.0)	n, %
SiRe® tested tissue samples	SiRe® tested tissue samples	48, 96.0
*KRAS*	Total mutations	1, 2.0
	Alone	1, 2.0
*KRAS* mutations	Exon 2 non p.G12C	1, 100.0
*EGFR*	Total mutations	0, 0.0
*EGFR* mutations	–	–
*BRAF*	Total mutations	18, 37.5
	Alone	14, 29.2
	Co-occurring mutations with other tested genes	4, 8.3
*BRAF* mutations	Exon 15 non p.V600E	8, 44.4
	Exon 15 p.V600E	10, 55.6
*PIK3CA*	Total mutations	1, 2.0
	Co-occurring mutations with other tested genes	1, 2.0
*PIK3CA* mutations	Exon 9	1, 100.0
*NRAS*	Total mutations	14, 29.2
	Alone	12, 25.0
	Co-occurring mutations with other tested genes	2, 4.2
*NRAS* mutations	Exon 2	1, 7.1
	Exon 3	13, 92.9
*KIT*	Total mutations	3, 6.2
	Alone	2, 4.2
	Co-occurring mutations with other tested genes	1, 2.0
*KIT* mutations	Exon 11	3, 100.0
*PDGFRα*	Total mutations	0, 0.0
*PDGFRα* mutations	–	–

*BRAF* V-Raf murine sarcoma viral oncogene homolog B, *EGFR* Epidermal growth factor receptor; *KIT* KIT proto-oncogene, receptor tyrosine kinase, *KRAS* Kirsten rat sarcoma viral oncogene homolog, *n* Number, *NRAS* Neuroblastoma RAS viral oncogene homolog, *PDGFRα* Platelet derived growth factor receptor alpha; *PIK3CA* Phosphatidylinositol-4,5-bisphosphate 3-kinase catalytic subunit alpha

No mutations were reported in the n = 2 liquid biopsy samples.

### Breast cancer

Overall, the SiRe® panel analyzed 43 breast cancer samples (2.0%), achieving a 95.3% success rate (41/43). Most specimens were tissue samples (73.2%, 30/41), primarily histological (93.3%, 28/30) with surgical resections (64.3%, 18/28) and biopsies (35.7%, 10/28); the remainder were cytological smears (6.7%, 2/30) or plasma biopsies (26.8%, 11/41). Among the 30 adequate tissue samples, 46.7% (14/30) harbored at least one DNA-based alteration. *PIK3CA* mutations were the most frequent (26.7%, 8/30), while concomitant alterations included *EGFR* + *PIK3CA* (6.7%, 2/30), *KRAS* + *BRAF* + *PIK3CA* (6.7%, 2/30), and *KRAS* + *KIT* + *PIK3CA* (3.3%, 1/30). Results are summarized in Table 6.

**Table 6:** SiRe® results on breast cancer tissue samples

N (%)	41 (100.0)	n, %
SiRe® tested tissue samples	SiRe® tested tissue samples	30, 73.2
*KRAS*	Total mutations	3, 10.0
	Co-occurring mutations with other tested genes	3, 10.0
*KRAS* mutations	Exon 2 non p.G12C	2, 66.7
	Exon 2 p.G12C	1, 33.3
*EGFR*	Total mutations	2, 6.7
	Co-occurring mutations with other tested genes	2, 6.7
*EGFR* mutations	Exon 19 point mutations	2, 100.0
*BRAF*	Total mutations	3, 10.0
	Co-occurring mutations with other tested genes	3, 10.0
*BRAF* mutations	Exon 11	2, 66.7
	Exon 15 non p.V600E	1, 33.3
*PIK3CA*	Total mutations	13, 43.3
	Alone	8, 26.7
	Co-occurring mutations with other tested genes	5, 16.6
*PIK3CA* mutations	Exon 2	1, 7.7
	Exon 5	1, 7.7
	Exon 9	6, 46.2
	Exon 20	4, 30.7
	Co-mutations	1, 7.7
*NRAS*	Total mutations	0, 0.0
*NRAS* mutations	–	–
*KIT*	Total mutations	1, 3.3
	Co-occurring mutations with other tested genes	1, 3.3
*KIT* mutations	Exon 11	1, 100.0
*PDGFRα*	Total mutations	0, 0.0
*PDGFRα* mutations	–	–

*BRAF* V-Raf murine sarcoma viral oncogene homolog B, *EGFR* Epidermal growth factor receptor; *KIT* KIT proto-oncogene, receptor tyrosine kinase, *KRAS* Kirsten rat sarcoma viral oncogene homolog, *n* Number, *NRAS* Neuroblastoma RAS viral oncogene homolog, *PDGFRα* Platelet derived growth factor receptor alpha, *PIK3CA* Phosphatidylinositol-4,5-bisphosphate 3-kinase catalytic subunit alpha

As for the liquid biopsy samples, n = 3 (27.3%) patients displayed a *PIK3CA* mutation in exons 2, 5, and 9.

### Other tested samples

Overall, n = 8 (0.4%) out of 2173 tested samples derived from other tumor types. In particular, n = 1 from small cell carcinoma (SCLC) displayed a *KRAS* exon 2 p.G12D mutation, those from thyroid carcinoma (n = 5) displayed *KRAS* exon 3 p.Q61R and *NRAS* exon 3 p.Q61L mutations, those from penile SqCC (n = 1) displayed a *PIK3CA* exon 9 p.E545K mutation, and those from pancreas ADC (n = 1) displayed a *KRAS* exon 2 p.G12R mutation.

## Discussion

Personalized medicine has profoundly transformed the management of patients with advanced-stage solid tumors [4]. With the increasing number of actionable biomarkers for targeted therapies, next-generation sequencing (NGS) has become central in molecular predictive pathology laboratories [15]. This powerful technology allows cytopathologists to capture the molecular landscape of multiple actionable alterations in a single run, even when nucleic acid input is very low [10]. Up until December 2022, our predictive molecular pathology laboratory successfully implemented a DNA-based NGS approach to detect targetable alterations across various solid tumors, including NSCLC, CRC, GIST, melanoma, and breast cancer [10, 11].

In this study, we retrospectively analyzed data from advanced-stage solid tumor patients referred to our laboratory for molecular testing between January 2018 and December 2022. Overall, 2,173 patients underwent testing using our DNA-based SiRe® NGS panel. Notably, our failure rate due to inadequate results or rejected samples was only 5.9% (128/2,173). Most samples were from NSCLC patients (60.4%, 1,312/2,137), followed by CRC (31.8%, 691/2,173), GIST (3.1%, 68/2,173), melanoma (2.3%, 51/2,173), breast cancer (2.0%, 43/2,173), and other tumor types (0.4%, 8/2,173).

Our DNA-based SiRe® NGS panel successfully analyzed nearly all NSCLC samples (92.1%, 1,208/1,312), consistent with the low failure rate reported. Beyond NSCLC testing, the panel, in accordance with international guidelines [16], effectively analyzed various histological NSCLC subtypes. The majority of patients had NSCLC adenocarcinoma/favor adenocarcinoma (87.5%), followed by NSCLC NOS (5.7%), NSCLC squamous/favor squamous (5.5%), NSCLC adenosquamous (1.1%), and NSCLC LCNEC/favor LCNEC (0.2%). *KRAS* mutations were the most common. While *KRAS* mutations are generally negative prognostic factors [17, 18], identifying the exon 2 p.G12C mutation is critical for selecting patients eligible for specific *KRAS* exon 2 p.G12C inhibitors, such as adagrasib and sotorasib [19, 20].

*EGFR* was the second most frequently mutated gene. “Common” exon 19 deletions and exon 21 point mutations confer sensitivity to all generations of EGFR TKIs, but detecting “uncommon” alterations, including exon 18 and 20 point mutations and compound mutations, is crucial for predicting response to EGFR-targeted therapy [21–23]. For instance, *EGFR* exon 20 insertions have limited response to EGFR TKIs [21, 24] but show high sensitivity to the bispecific monoclonal antibody amivantamab [25, 26]. Similar to *KRAS*, *BRAF* mutations were historically negative prognostic markers in NSCLC. However, following the approval of dabrafenib plus trametinib for *BRAF* exon 15 p.V600E patients, *BRAF* now serves as a positive predictive marker in NSCLC [27, 28].

*PIK3CA* mutations were more frequent in NSCLC squamous/favor squamous tumors, consistent with literature reports [29], and may be highly sensitive to specific inhibitors such as alpelisib, even when occurring alone [30]. *NRAS* mutations were rare (< 1% of NSCLC cases), but emerging evidence suggests potential benefit from chemo-immunotherapy in these patients [31].

In addition to DNA-based biomarkers, actionable alterations in NSCLC such as *ALK*, *ROS1*, *RET*, *NTRK* rearrangements, *MET* exon 14 skipping, *HER2* mutations, and PD-L1 expression are strongly recommended by international guidelines [16]. However, in our experience, referrals for these molecular tests were fragmented, with only 52.7% (692/1,312), 50.8% (667/1,312), and 50.7% (665/1,312) of NSCLC patients referred for ALK, ROS1, and PD-L1 testing, respectively. This fragmentation, likely due to testing being performed external at referring centers, can limit comprehensive molecular assessment and tissue availability [32]. We therefore recommend that all molecular testing be performed simultaneously in the same laboratory. RET, NTRK, and MET testing were even less frequent (15.6%, 205/1,312), likely reflecting late drug approvals in 2022 [16]. In addition, a low prevalence of *ALK* rearranged and *MET* exon 14 skipping cases was observed in our series. We postulate that this finding may be attributable to stochastic variation rather than to limited detectability of the employed assays (IHC/ICC or RNA-based NGS). Notably, the introduction of RNA-based NGS in January 2021 has enabled us to optimize sample utilization for the simultaneous analysis of clinically relevant biomarkers, while reducing turnaround time and preserving valuable material for additional testing (e.g., IHC/ICC for PD-L1 expression evaluation).

Liquid biopsy also proved highly effective for NSCLC mutation detection, especially in cases with limited tissue. In our cohort, 37.0% (447/1,208) of NSCLC samples were liquid biopsy specimens, highlighting its value for both baseline and resistance testing [33–36].

Metastatic colorectal cancer (mCRC) was the second most analyzed tumor type (31.8%, 691/2,173), with 97.1% (671/691) of samples successfully analyzed by our panel. Tissue samples were most commonly used (96.1%, 645/671). *KRAS* was the most frequent mutation and, together with *NRAS*, historically predicted poor response to anti-EGFR therapy [37]. As in NSCLC, *KRAS* exon 2 p.G12C now guides selection for sotorasib or adagrasib [38, 39]. *BRAF* exon 15 p.V600E predicts sensitivity to encorafenib, binimetinib, and cetuximab combination therapy, improving overall survival [40–42]. *PIK3CA* mutations often co-occur with *RAS*/*BRAF* alterations but may also predict response to specific PIK3CA inhibitors [43]. *EGFR* and *KIT* mutations were rare in mCRC [44]. MSI or dMMR status, critical for immunotherapy selection, was tested in only 56.7% (366/645) of cases, likely due to testing at primary centers. We recommend performing MSI/dMMR assessment alongside other molecular tests for accurate treatment decisions, as supported by KEYNOTE-177 data [45]. Liquid biopsy also plays a growing role in guiding anti-EGFR rechallenge therapy in mCRC [46, 47].

GIST was the third most analyzed tumor type (3.1%, 68/2,173), with a successful analysis rate of 98.5% (67/68). Most patients harbored KIT mutations, which predict response to imatinib, and PDGFRα alterations, which also guide TKI therapy. Rare BRAF exon 15 p.V600E and EGFR mutations were detected, with liquid biopsy enabling continuous monitoring [48–54].

Melanoma accounted for 2.3% (51/2,173) of cases, with a 98.0% success rate for our panel. *BRAF* exon 15 p.V600E mutations were most common and critical for TKI therapy, while *NRAS* and *KIT* mutations, more frequent in non-cutaneous melanomas, provided additional predictive information [55–59]. Liquid biopsy is increasingly applied in melanoma management.

Breast cancer represented 2.0% (43/2,173) of cases, with 95.3% (41/43) successfully analyzed. *PIK3CA* mutations were most frequent and predictive of alpelisib efficacy in ER-positive/HER2-negative advanced-stage patients [60]. Other mutations (*KRAS*, *EGFR*, *KIT*, *BRAF*) were rare, and liquid biopsy is gaining traction for prognostic and predictive applications [61, 62].

In conclusion, this study provides real-world evidence on the efficiency of DNA-based NGS in tissue and liquid biopsy for detecting actionable mutations in advanced-stage solid tumors. While small gene NGS panels remain common in Italy, our SiRe® panel, although limited to seven genes, is analytically validated and routinely implemented for both tissue and liquid biopsy and provides reliable detection of clinically relevant mutations [10]. Limitations include fragmented genomic testing and restricted gene panel, single-institute data, the retrospective nature of the analysis, lack of external or multicentre validation, heterogeneity of tissue samples, and the absence of clinical data on the impact of the detected mutations on patient management and clinical outcome. Another important point is that this experience was conducted using a small panel covering only seven genes. This choice reflects the fact that, at the time the molecular testing was performed, these were the clinically relevant genes for the analyzed solid tumors. However, a major limitation was the lack of information on other mutated genes, such as tumor protein 53 (*TP53*), which may be useful for oncologists in clinical decision-making. In addition, it should be noted that several molecular findings were interpreted in light of current drug approvals, some of which occurred during or after the study period. These interpretations are therefore retrospective and reflect current therapeutic options rather than real-time treatment decisions made between 2018 and 2022. Another limitation is the absence of molecular analyses for central nervous system malignancies, as these tests are centralized at another institution. Further studies using broader NGS panels in tissue and liquid biopsies with information regarding clinical data are warranted to fully explore the complex genomic landscape of advanced-stage solid tumors [9, 63–67]. Looking forward, future research will focus on validating these findings through prospective multicentre studies designed to assess reproducibility, analytical robustness, and inter-laboratory concordance. Additionally, integrating matched clinical outcome data will be essential to evaluate the real-world clinical utility of detected alterations, including response to targeted therapies, progression-free survival, and overall survival. The authors also plan to expand the molecular workflow by incorporating RNA-based assays, broader comprehensive NGS panels, and standardized reflex testing strategies to overcome current fragmentation in diagnostic pathways. These steps will be crucial to establish the full clinical impact of NGS analysis, strengthen its adoption in routine practice, and ensure that patients with advanced-stage solid tumors receive the most accurate and timely precision oncology interventions.

## Supplementary Information

Below is the link to the electronic supplementary material.

Supplementary file1 (DOCX 12 kb)

Supplementary file2 (DOCX 17 kb)

Results obtained on all analyzed samples. Abbreviations: NT, not tested. Supplementary file3 (XLSX 394 kb)

## Data Availability

The datasets generated during and/or analysed during the current study are available from the corresponding author on reasonable request.
