# Supplementary material for: Next-generation sequencing using a targeted gene panel in advanced solid tumors: five years of experience from an Italian referral institution
Source: Clin Transl Oncol. 2026 Jun 3;28(11):5462–78. doi: 10.1007/s12094-026-04374-w (PMC13694439; doi:10.1007/s12094-026-04374-w)
Supplement: Supplementary file 1 [file 12094_2026_4374_MOESM1_ESM.docx]

**Supplementary Table 1.** Variant caller parameters file for variant calling plug in 5.0

| Parameters | SNP | INDEL | HotSpot |
| --- | --- | --- | --- |
| Minimum allele frequency | 0,1 | 0,1 | 0,0001 |
| Minimum quality | 20 | 20 | 20 |
| Minimum coverage | 5 | 5 | 5 |
| Minimum coverage on either strand | 0 | 0 | 0 |
| Maximum strand bias | 1 | 1 | 1 |
| Minimum relative read quality | 5 |  |  |
| Maximum common signal shift | 0,3 |  |  |
| Maximum reference/variant signal shift (insertions) | 0,3 |  |  |
| Maximum reference/variant signal shift (deletions) | 0,3 |  |  |

Clinical sensitivity: Run median output was 257Mbases, median read length was 124 bp, mean read depth was 2821X and coverage uniformity was 97%.[ref. 11]
