# Supplementary material for: Next-generation sequencing using a targeted gene panel in advanced solid tumors: five years of experience from an Italian referral institution
Source: Clin Transl Oncol. 2026 Jun 3;28(11):5462–78. doi: 10.1007/s12094-026-04374-w (PMC13694439; doi:10.1007/s12094-026-04374-w)
Supplement: Supplementary file 2 [file 12094_2026_4374_MOESM2_ESM.docx]

| **Supplementary Table 2**. SiRe® reference range. | | |
| --- | --- | --- |
| Gene | Exon | Mutations |
| EGFR | 7 | A289T; A289D; A289V |
|  | 18 | A698T; A702S; E709K; E709H; E709V; E709G; E709A; F712L; F712S; G719C; G719S; G719D; G719A; G721S; G721A; G724S; L703P; S720F |
|  | 19 | A743T; A750P; D761N; D761Y; D761G; E734K; E746_E749delELRE; E746_A750delELREA; E746_T751delELREAT; E746K; E746_S752delELREATS; E746_T751>A; E746_T751>V; E746_S752>A; E746_S752>V; E746_S752>D; G735S; K745_E749delKELRE; K745R; K754E; L730F; L747_T751delLREAT; L747P; L747_E749delLRE; L747_A750>P; L747_T751>P; L747_S752delLREATS; L747_P753>S; L747S; L747_T751>S; L747_P753>S; L747_R748>FP; P733S; P733L; P741L; P753S; S752_I759delSPKANKEI; S752P; S752Y; T751I; T751_E758delTSPKANKE; T751_I759>N; V738G; V742A; W731* |
|  | 2 | A767V; C775Y; D770N; D770>GY; D770_N771insG; D770_N771insSVD; G779F; G810S; G810D; H773L; H773R; H773_V774insH; H773_V774insNPH; I821T; L792P; L814P; N771_P772insN; R776C; R776H; S768I; S784P; S784F; V765M; V769L; V769_D770insASV; V774M; V786M; V819V |
|  | 21 | A859T; A864T; A871T; A871G; E868G; G863D; G873E; G874S; H870Y; H870R; L858M; L858L; L858R; L861R; L861Q |
|  | 15 | G598V; P596L |
|  | 3 | R108K |
|  | 20 | T790M |
| KIT | 2 | D52N; G51G |
|  | 11 | D572N; D579del; E554K E554_K558del; E561K; E561E; E562K; F584S; I563_L576del; I571_L576del; K558E; K558_V559>SS; K558_V559del; K558_V560del; K558_E562del; K558_N564del; K558R; K558_V560>I; K558K; K558>NP; K558_V560>N; L576F ; L576del; L576P; M552_W557del; M552L; M552_Y553del; M552_E554del; M552_Q556del; M552_E554>K; M552_Q556>K; N564_L576del; N564_Y578del; N566D; N566N; N567K; P551_V555del; P551L; P573L; P577S; P585P; Q556_K558del; Q556_V560del; Q556_L576del; Q556_V560>H; V555_E562del; V555I; V555_Q556del; V555_K558del; V555_V559del; V555_V560del; V555_I571del; V555_P573del; V559I; V559del; V559_V560del; V559_E561del; V559_G565del; V559D; V559G; V559A; V560del; V560_L576del; V560D; V560G; V560E; W557_K558del; W557_E561del; W557R; W557G; W557del; W557_K558>E; W557_V559del; W557_V560del; W557_E561del; W557S; W557_V559>F; W557_P573>S; W557_K558>CP; W557_V559>C; W557_V560>C; W582*; Y553_W557del; Y553_K558>; Y553N; Y553_Q556del; Y553_K558del; Y553_T574>S; Y568D; Y568_T574del; Y570_L576del |
|  | 17 | D816Y; D816H; D816F; D816I; D816G; D816V; D816E; D820Y; D820H; D820G; D820V; D820E; F811F; K818R; N822Y; N822K; V825I; V825A; Y823D; Y823C |
|  | 18 | E839K; W853* |
|  | 9 | K509I; Y503_F504insAY |
|  | 10 | M541L; V530I; K546K |
|  | 13 | K642E; V643A; V654A |
|  | 15 | S715del |
|  | 14 | T670E; T670I |
| PDGFRA | 18 | D842Y; D842I; D842delD; D842_M844delDIM; D842_D846>N; D842V; D842_D846>E; I843_D846delIMHD; I843_S847>T; M844_S847delMHDS; D846Y; R841_D842delRD; V824V |
|  | 14 | N659Y; N659K |
|  | 12 | R554_Y555delRY; R560_S564delRVIES; S566fs*6; S566_E571>K; S566_E571>R; V561D |
|  | 15 | T674I |
| KRAS | 2 | A11_G12insGA; A11V; A11P; A18D; G12_G13insG; G12G; G12D; G12V; G12E; G12W G12C; G12V; G12D; G12A; G12Y; G12I; G12F; G12L; G12C; G12S; G12R; G10_A11insG; G13_V14insG; G13G; G13V; G13D; G13E; G13R; G13A; G13C; G13S; G13R; G15S; Q22K; L19F; V14I; V8V |
|  | 3 | A59G; A59E; A59T; Q61H; Q61P; Q61R; Q61L; Q61K; Q61E; T58I |
|  | 4 | A146V; A146T; A146P; K117N |
| NRAS | 2 | A11T; A18T; G12G; G12D; G12A; G12V; G12N; G12R; G12C; G12S; G13G; G13V; G13D; G13A; G13R; G13C; G13S |
|  | 3 | G60E; Q61H; Q61Q; Q61L; Q61R; Q61K; Q61P; Q61E; S65C |
|  | 4 | A146T |
| BRAF | 15 | A598_T599insV A598V; D587E; D587A; D594E; D594V; D594G; D594N; E586E; E586K; F583F; F595L; F595S; G596D; G596R; G606E; H608R; K601N; K601del; K601E; I592M; I592V; L584L; L584F; I582M; L597L; L597Q; L597R; L597S; L597V; N581S; S605N; S605F; S605G; R603*; T599_V600insTT; T599_V600insT; T599I; V471F; W604G; W604del; V600_K601>E; V600E; V600D; V600A; V600G; V600Q; V600K; V600R; V600M; V600L |
|  | 11 | G464E; G464V; G464R; G466V; G466A; G466E; G466R; G469S; G469V; G469A; G469E; G469R; P453T; R462I; R444W |
| PIK3CA | 2 | R88Q |
|  | 5 | I391M; N345K |
|  | 7 | p.V461_N465del; C420R; p.L445CfsTer9 |
|  | 9 | G685D; G686M; Q682H; P539R; Q546E; E542K; E545Q; E542V; E545A; E545G; E545K; Q546K; Q546P; Q546R; E545D |
|  | 20 | H1047L; G1049L; G1049S; H1047R; H1047Y; M1043I |
